# Haptic Discrimination of Distance

**DOI:** 10.1371/journal.pone.0104769

**Published:** 2014-08-12

**Authors:** Femke E. van Beek, Wouter M. Bergmann Tiest, Astrid M. L. Kappers

**Affiliations:** MOVE Research Institute Amsterdam, Faculty of Human Movement Sciences, VU University, Amsterdam, The Netherlands; Emory University, United States of America

## Abstract

While quite some research has focussed on the accuracy of haptic perception of distance, information on the precision of haptic perception of distance is still scarce, particularly regarding distances perceived by making arm movements. In this study, eight conditions were measured to answer four main questions, which are: what is the influence of reference distance, movement axis, perceptual mode (active or passive) and stimulus type on the precision of this kind of distance perception? A discrimination experiment was performed with twelve participants. The participants were presented with two distances, using either a haptic device or a real stimulus. Participants compared the distances by moving their hand from a start to an end position. They were then asked to judge which of the distances was the longer, from which the discrimination threshold was determined for each participant and condition. The precision was influenced by reference distance. No effect of movement axis was found. The precision was higher for active than for passive movements and it was a bit lower for real stimuli than for rendered stimuli, but it was not affected by adding cutaneous information. Overall, the Weber fraction for the active perception of a distance of 25 or 35 cm was about 11% for all cardinal axes. The recorded position data suggest that participants, in order to be able to judge which distance was the longer, tried to produce similar speed profiles in both movements. This knowledge could be useful in the design of haptic devices.

## Introduction

Humans often have to perceive to which location they move their arm, for instance when reaching for the light switch in the dark. Usually, these kinds of tasks are a combination of the perception of distance and position [1], [2], [3], [4]. In general, reproducing a position yields a movement ending closer to the physical location than reproducing a distance [5], [6], [7]. The former, perception of position, has received a lot of attention in, for instance, work on the motor system (e.g. [8], [9]). In this article, we focus on the latter, the haptic perception of distance. Distance can be perceived in two different ways. Firstly, it can be perceived by exploring the length of a hand-held object. For this type of exploration, the finger span-method is often used, which involves the perception of the length of an object that is pinched between the thumb and index finger [10], [11], [12], [13]. Secondly, distance can be perceived by moving the hand over a certain distance, which involves tracing an object or moving over a well-defined path between a start and end position [5], [7], [14], [15]. Like all forms of measurements, perception can be described using the terms perceptual *accuracy* (also called constant error or bias) and *precision* (also called random error or discrimination threshold). Most studies on haptic distance perception have been focussed on perceptual accuracy, while precision has received hardly any attention, especially in the case of the movement method. In this article, we would like to extend the knowledge on the precision of distance perception using the movement method. Precise arm movements can be very important in, for instance, applications like haptic devices. Moreover, from a fundamental point of view, knowledge on perceptual precision provides a measure of the repeatability of the data obtained in these kinds of experiments. This can be a valuable addition to data on perceptual accuracy, which describe biases in perception.

We try to answer four main questions, which are: what are the effects of reference distance, movement axis, movement mode (active or passive), and stimulus type on the precision of haptic perception of distance? In the following paragraphs the existing knowledge on the effects of the four conditions on both aspects (accuracy and precision) of haptic perception of distance will be described.

### Reference distance

For the finger-span method, which can be used to perceive distances of hand-held objects, the relation between physical length and perceived length is a power function with an exponent ranging between 1.1 and 1.3 [16], [17], [18], [19]. For the movement method, a power function with an exponent of about 0.89–1 is reported [20], [21]. In general, short distances are underestimated and long distances are overestimated (e.g. [22]).

The precision of length perception as a function of reference length has been studied for the finger-span method. Gaydos [23] reports a stable Weber fraction (Wf: the smallest perceivable difference - also called discrimination threshold - divided by the stimulus intensity) of about 4% for reference lengths larger than 35 mm. Below that length, the Weber fraction increases up to 10% for a reference length of 10 mm [10], [11], [19]. However, nothing is known about the influence of reference distance on the precision of haptic distance perception using the movement method.

### Movement axis

For the accuracy of haptic distance perception using arm movements, an anisotropy between movements along different axes exists. This anisotropy is called the radial-tangential or the horizontal-vertical illusion (for a review, see Gentaz and Hatwell [24], [25]) and it entails that the radial (vertical) segment of the figure is perceived as longer than the tangential (horizontal) segment [15], [26], [27]. There have been numerous studies and debates concerning the influence of particular task characteristics on the presence and size of the effect. Generally, it is found that a distance is overestimated (underestimated) when the arm is moved at a slower (faster) speed [28], [29], [30] and as radial movements are indeed executed slower, this might be an explanation for the anisotropy [15], [26]. Recently, however, McFarland and Soechting [31] systematically manipulated arm speed and effort of participants judging radial and tangential distances and found no effect of either manipulation on the size of the illusion. Other authors have shown that the illusion is still present when the L-shape is presented at an angle with the radial and tangential axes [32], [33], [34], but it disappears when the stimulus is presented in the vertical (fronto-parallel) plane [34], [35].

The question remains whether this anisotropy is also present in the precision of distance perception. Apart from early work, presenting data of only one subject for the comparison of 2 distances [30], [36], no data on precision along different movement axes is available.

### Movement mode

Distance can be perceived either passively, by being guided over a distance or by moving a surface under a stationary finger [14], [31], or actively, by exploring the length of a hand-held object [12], [13] or by moving over a certain distance [5], [7], [15]. It seems that active perception provides a more accurate percept than passive perception [3], with the movement distance being slightly underestimated in the passive case [37], [38]. For a review on the difference between active and passive perception, see Symmons et al. [39].

Again, it is not known what the effect on precision is. This comparison could provide insight in the question whether distance perception is purely based on the start and end position of the movement, or also on the way in which the movement itself is made.

### Stimulus type

Some work on perceptual accuracy for different types of haptic stimuli has been performed. Noll and Weber [40] found that distances are considerably underestimated when perceived purely cutaneously by moving a medium under the finger. When the finger is moved over the medium, thus providing cutaneous and kinaesthetic cues, the underestimation is much smaller. Terada et al. [41] added a condition with only kinaesthetic cues and found the underestimation to be in-between that of the former two conditions for distances of 100 and 150 mm. Conversely, Van Doorn et al. [42] report that for a stimulus length of 40 mm subjects were more accurate at judging line length when using cutaneous cues alone, compared to using kinaesthetic cues or a combination of the two.

Recently, Bergmann Tiest et al. [14] performed discrimination experiments that involved passive perception of distance by moving the hand over a distance, moving a surface under the static finger, and moving the finger over a static surface, all over a distance of 80 mm. They found Weber fractions of 25% for the stimulus moving under the finger and 11% for the other two conditions. It therefore seems that distance perception is possible when purely cutaneous information is present, but it improves when kinaesthetic information is added. However, the combination does not seem to be better than kinaesthetic information alone. It is still unknown what the contribution of the different cues in an active situation would be.

There are not many studies in which the distance perception of both real and rendered stimuli have been tested. One study reports that the precision of perceiving distances using a stylus to probe a surface is slightly better in a real than in a simulated environment [43]. Whether there is still a difference in perception when a stylus is not used, is unknown.

From the studies mentioned above it is apparent that the precision of haptic distance perception is still a largely unexplored area. In our study, we investigated the effects of movement axis, stimulus type, movement mode and reference distance on this precision.

## Materials and Methods

### Participants

Twelve naive participants took part in this study, 5 male and 7 female, aged 22±3 years (mean±standard deviation), with no known neurological disorders. Handedness was assessed using the Coren-test for handedness [44], which confirmed that all participants were right-handed. All participants gave written informed consent to participate in the study and received a small compensation for their time. Prior to the experiment, they were given written instructions on how to perform the experiment. This experiment was approved by the Ethics Committee of the Faculty of Human Movement Sciences (ECB).

### Conditions

**Figure 1:**
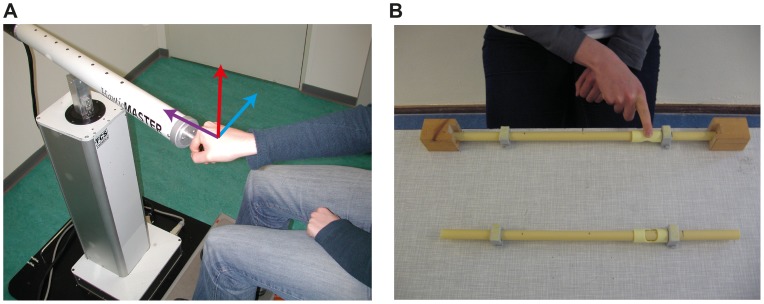
Pictures of the setups used in the experiment. **A**. The haptic device used for the conditions involving rendered stimuli, which were conditions 1 through 6. The three movement axes are indicated with arrows, light blue is the tangential, purple the radial and red the vertical axis. **B**. The setup used for the conditions involving real stimuli, which were conditions 7 and 8. During the experiment, markers were taped on top of the blocks and on the nail of the participant's right index finger. These markers are not shown in this picture. The participant demonstrates condition 7, in which the finger is placed on the closed side of the carriage. The carriage at the other tube is placed with the open side up, as used in condition 8.

Eight conditions were measured in this experiment. The baseline condition was active distance perception, using the handle of a haptic device. In this condition, the reference distance was 25 cm, the movement axis was tangential to the participant in the horizontal plane and the haptic cues were of a kinaesthetic nature. The other conditions were variations in reference distance, movement axis, perceptual mode and stimulus type. The reference distance was either 15, 25 or 35 cm. The movement axis was either tangential, radial or vertical to the participant (see Fig. 1A). The perceptual mode was either active or passive. The type of stimulus was either rendered kinaesthetic, real kinaesthetic or real kinaesthetic+cutaneous. For an overview of all conditions, see Table 1.

**Table 1:** Experimental conditions.

Condition	Reference distance	Movement axis	Mode	Stimulus type
1	25 cm	tangential	active	rendered
2	15 cm	tangential	active	rendered
3	35 cm	tangential	active	rendered
4	25 cm	radial	active	rendered
5	25 cm	vertical	active	rendered
6	25 cm	tangential	passive	rendered
7	25 cm	tangential	active	real
8	25 cm	tangential	active	real+cutaneous

Overview of all the experiment conditions. Each column represents one research question.

### Setup

The setup used for the rendered stimuli (conditions 1 through 6 in Table 1) was a 3 degrees of freedom haptic device, the Haptic Master (Moog Inc.). This is an admittance-controlled device, so the device is capable of simulating very stiff virtual objects. A virtual tunnel that was 2×2 mm wide was simulated, within which the participants could move a probe freely. The probe was a virtual point, which was located in the center of a ball-shaped handle (42 mm diameter). The handle was connected rigidly to the haptic device through a metal bar. Subjects were instructed to grab the handle always in such a way that the metal bar was positioned between their index and middle finger and their palm was resting on top of the ball-shaped handle. For a top view of a participant holding the handle of the haptic device, see Fig. 1A. Participants could not move the probe out of the tunnel, as simulated walls with a stiffness of 20 kN/m prevented this. The length of the tunnel determined the movement distance. The position of the tunnel in space determined the start and end position. The orientation of the tunnel determined the movement axis. In the active condition, participants were asked to move their arm from the start to the end of the tunnel themselves. In the passive condition, the haptic device moved the arm of the participants.

The movement distances for conditions 1 through 6 were: a reference distance of 25 cm, and test distances of 21, 22, 23, 24, 26, 27, 28 and 29 cm. For the reference distances of 15 and 35 cm, the test distances ranged between 11 and 19 cm and between 31 and 39 cm, respectively, in steps of 1 cm. The method of constant stimuli was used, in which all test distances were presented 10 times for every condition, resulting in a total of 80 trials per condition. In all conditions, the start positions were offset either −2, 0 or +2 cm. The offset was assigned in a pseudo-random manner. The start position always differed between the first and the second movement of one trial.

Participants were seated on a 62 cm high chair, while their arm height was 95 cm. In the tangential condition, the mean position halfway between the two stops was in front of the participant's sternum, while he or she made a movement from left to right. In the radial condition, the height of the arm was the same, but the movement was along the radial axis in front of the sternum. In the vertical condition, the movement started at approximately the height of the other two conditions and was made upwards, again in front of the sternum. For an overview of the setup for the rendered conditions and the movement axes, see Fig. 1A.

For the conditions that involved real stimuli instead of rendered ones (conditions 7 and 8 in Table 1), another setup was used. This setup consisted of 27 PVC tubes with two stops each in between which a carriage could be moved (see Fig. 1B). The carriage had two sides, one open and one closed, to create two different types of stimuli. The closed side of the carriage, which was used in condition 7, prevented the participants from feeling the surface sliding underneath their finger during the trial. In condition 8, the participant inserted his or her finger in a hole in the open side of the carriage to slide it over the surface of the tube while moving from start to end position. In both real conditions the arm movements, seat height, and arm height were the same as in the baseline condition. In conditions 1 through 7, the only types of relevant haptic cues were kinaesthetic. Therefore, the only difference between the rendered baseline condition and condition 7 was the hand posture (moving a handle vs. using the index finger to slide a carriage) and the type of stimulus (rendered vs. real). In condition 8, conversely, participants could also use cutaneous information.

During the trials of the conditions using rendered stimuli, hand position was measured using the position measurement function of the Haptic Master, which tracked the position of the probe, located at the center of the ball-shaped handle. In the conditions using real stimuli, the position of the finger was measured with a TrakSTAR device (Ascension Technology Corporation), which tracked the position of a marker on the nail of the right index finger using a magnetic field transmitter. Two extra markers were placed at the end blocks of the setup, to facilitate the data analysis. Both devices sampled position with a frequency of 90 Hz.

### Procedure

Participants were blindfolded during the experiment. They participated in four one-hour sessions. During every session, two conditions were measured. At the start of every condition, three practice trials were performed. Every trial consisted of the comparison of two distances by moving once from start to end stop for every distance. During the movement, white noise was played on headphones worn by the participants to mask the sound of the device. A two-alternative forced-choice paradigm was used, so participants were only allowed to answer with ‘1’ or ‘2’ to indicate which distance they perceived to be the longer. Participants were provided with feedback on their answer, to direct them towards judging distance rather than position. Because the start position always differed between the two distances within one trial, participants could not use position cues directly and were forced to estimate distance. The order of start position and test distances was chosen pseudo-randomly and conditions were blocked. The order of the condition blocks was also chosen pseudo-randomly. Between conditions, participants were allowed to take a short break. Depending on the condition (see Table 1 for an overview), participants were asked to perform the task in a specified manner, as described below:

- Conditions 1 through 3: Participants moved the handle of the haptic device from left to right. At the right stop, they released the handle, after which it moved to the new start position. A sound and the start of the white noise indicated that participants could start a new movement. They again moved the handle from left to right and then indicated verbally which of the two distances they perceived to be the longer, after which a new trial began.
- Condition 4: Participants moved the handle from proximal to distal. The rest of the procedure was the same as for conditions 1 through 3.
- Condition 5: Participants moved the handle from the most downwards to the most upwards position. The rest of the procedure was the same as for conditions 1 through 3.
- Condition 6: After grabbing the handle, participants did not move it to the right themselves, but were moved by the device to the end position. The two distances within one trial were travelled with the same speed during half of the trials and had the same duration during the other half of the trials, in a pseudo-randomly assigned order. Mean movement speed was 0.167 m/s, mean movement duration was 90 seconds. The rest of the procedure was the same as for conditions 1 through 3.
- Conditions 7 and 8: After switching on the white noise, the experimenter placed the finger of the participant on the surface of the carriage (condition 7) or in the hole in the carriage (condition 8). The participant then moved the carriage until it hit the end stop. The experimenter replaced the rail with a rail with another distance, placed the carriage at the start position and switched the white noise on again, to indicate the start of the second part of the trial. The rest of the procedure was the same as for conditions 1 through 3.

### Data analysis

For each participant and condition, a psychometric curve was fitted to the answers of the participants, using a least-squares fitting procedure on the following equation:
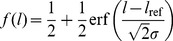
(1)


**Figure 2:**
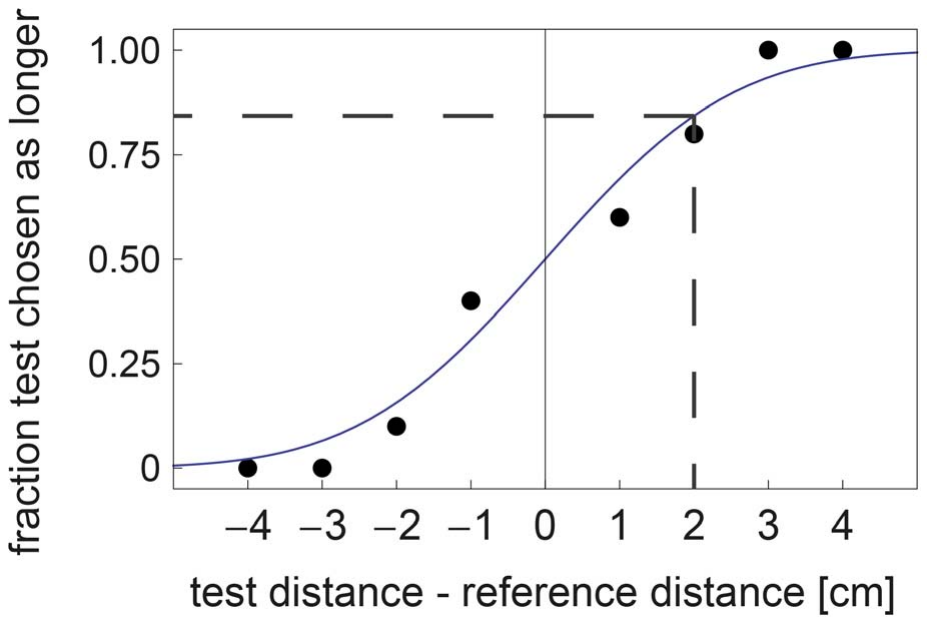
Example of a psychometric curve fitted to the data of a single participant in a single condition. Black points represent mean values of ten trials with the same test distance. The curve is fitted using the error function described in Eq. 1. Note that this function forces the fraction to be 0.5 when the test distance equals the reference distance. The discrimination threshold is the fitted 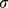 in the function, which is the value on the horizontal axis that corresponds to a fraction of 0.84. For the data in this figure, the fitted 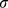 is 1.98 cm, which is indicated by the dashed lines.

For a typical example of such a fit to the data, see Fig. 2. The 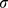 in this equation corresponds to the difference between the 0.50 and the 0.84 point, which is the discrimination threshold that was used for further analysis. To calculate the Weber fraction, the discrimination threshold was divided by the reference distance.

To investigate the effect of the various conditions on discrimination, a repeated measures ANOVA was performed per research question. This resulted in four ANOVAs for distance, movement axis, mode, and stimulus type, with condition as the within-subject factor. When the sphericity criterion was violated, Greenhouse-Geisser correction was used. Because the data set of the baseline condition was used in each of the four ANOVAs, the 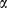 was Bonferroni-corrected by dividing it by 4, so a *p*-value smaller than 0.0125 was deemed significant in this procedure. When there was a significant main effect, post-hoc comparisons were performed using Bonferroni-correction. The corrected 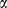 was based on the total number of post-hoc tests that were performed, which was 4, so a *p*-value smaller than 0.0125 was deemed significant for the post-hoc tests.

The data of the passive condition were further analyzed by dividing each set into a set with trials with the same movement speed and a set with the same movement time. To these two data sets per participant, new psychometric curves were fitted. The acquired thresholds were compared using a paired *t*-test.

**Figure 3:**
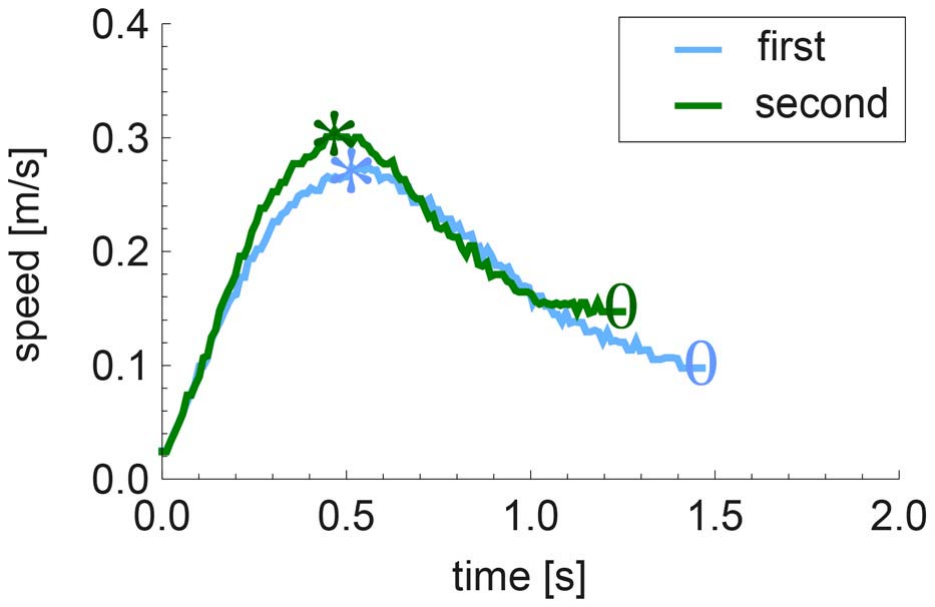
Typical example of speed data of the first (blue) and the second (green) movement of one trial. The asterisks show the moment of peak speed, while ovals indicate the end of the movement. For each trial, the horizontal (for time) and vertical (for speed) distances from the axes to the asterisks and ovals were calculated to determine the speed parameters. The speed difference parameters were then calculated by subtracting each parameter of the first trial from that of the second trial. Data collection was stopped immediately when the participant reached the end position, therefore the end speed is not zero.

From the position data, velocities were calculated for all active conditions. These were low-pass filtered using an 11-sample moving average, which yielded speed profiles like the example shown in Fig. 3. From these velocity profiles, the peak speed and end speed and their moment in time were calculated. This yielded the following parameters: peak speed, time of peak speed, end speed, and time of end speed. Within each trial, the difference between the parameters was calculated by subtracting the parameters of the first movement from those of the second. These difference parameters were then divided into two groups, based on the answer that the participant had given to the question which distance was the longer. For each condition and participant, the values were averaged over all trials. From these means per condition, a mean per participant per group was calculated for each parameter. The difference between the groups was assessed per parameter using a paired *t*-test. For one of the participants there was a measurement error in the position data of condition 7. The data of this condition were therefore not analyzed for this participant. One other participant was found to consistently have started his movement a little before the sound had indicated that he could start his trial. Because the data recording started simultaneously with the sound, the position for the first part of each trial was not recorded for this participant. Therefore, the position data of this participant were not used in the analysis. Overall, the position parameters were thus based on the mean value of 6 conditions for one participant and on the mean values of 7 conditions for the remaining 10 participants.

## Results

**Figure 4:**
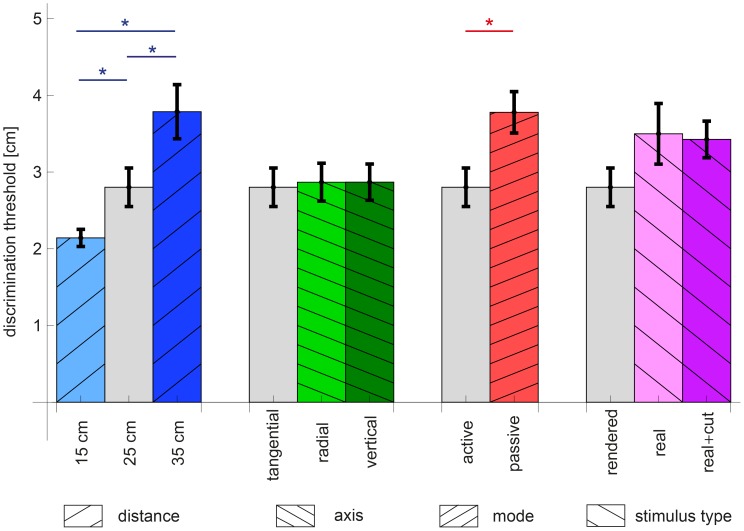
Discrimination thresholds, grouped according to research question. The bars denote the mean of all participants and the error bars show the standard error of the mean over participants. The labels at the horizontal axis indicate the measured conditions. Note that in each group the grey bar without hatching represents the baseline condition. See Table 1 for an explanation of all the conditions. * 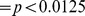 (Bonferroni-corrected 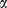)

**Figure 5:**
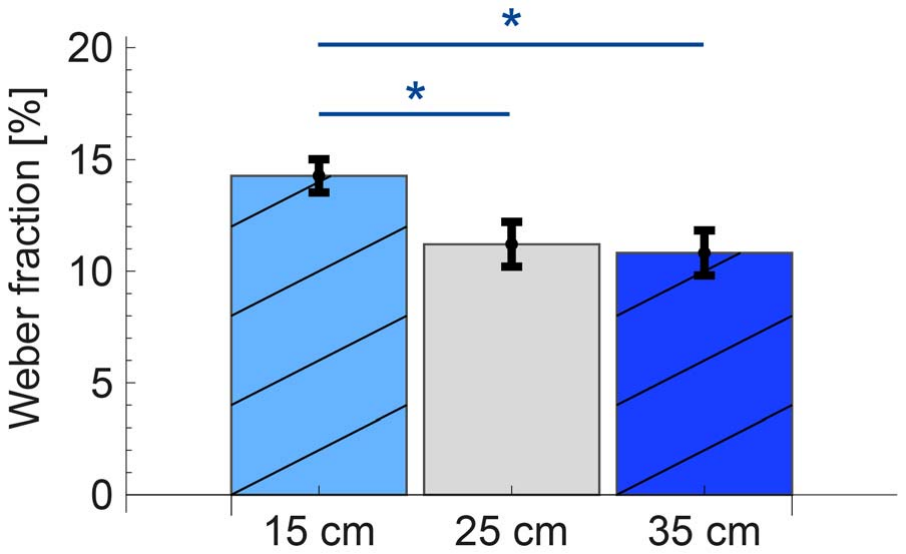
Precision for the three reference distances, represented as Weber fractions. The Weber Fraction is calculated by dividing the discrimination threshold by the reference distance. The bars show the mean over participants and the error bars show the standard error of the mean over participants. The Weber fraction for 15 cm is slightly larger than the fractions for 25 and 35 cm. * 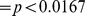 (Bonferroni-corrected 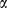)

An overview of the discrimination thresholds for all conditions can be found in Fig. 4. Each group of bars in the figure answers one of the research questions. The ‘distance’ group showed mean thresholds of 2.1±0.1, 2.8±0.3 and 3.8±0.4 cm (mean±standard error of mean) for a reference distance of 15, 25 and 35 cm, respectively. The ANOVA showed a significant main effect of condition (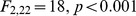). Post-hoc comparisons showed that all conditions within this group differed significantly (15 to 25 cm: 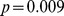, 25 to 35 cm: 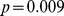, 15 to 35 cm: 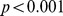). When expressed as Weber fractions by dividing the thresholds by the reference distances, the fractions for the ‘distance’ group were 14.3±0.7, 11.2±1.0, and 10.8±1.0% for a reference distance of 15, 25 and 35 cm, respectively (see Fig. 5). In this case, there was a main effect of reference distance (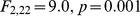), but only the Weber fraction for a reference distance of 15 cm was significantly different from those for 25 and 35 cm (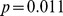 and 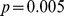, respectively). The ‘axis’ group showed mean thresholds for tangential, radial and vertical movements of 2.8±0.3, 2.9±0.2 and 2.9±0.2 cm, respectively. These thresholds were not significantly different (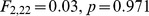). The ‘mode’ group showed mean thresholds of 2.8±0.3 and 3.8±0.3 cm for the active and the passive condition, respectively. These thresholds were significantly different (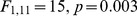). The ‘stimulus type’ group showed mean thresholds of 2.8±0.3, 3.5±0.4 and 3.4±0.2 cm for the rendered, real and real+cutaneous condition, respectively. These thresholds were not significantly different (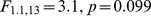).

**Figure 6:**
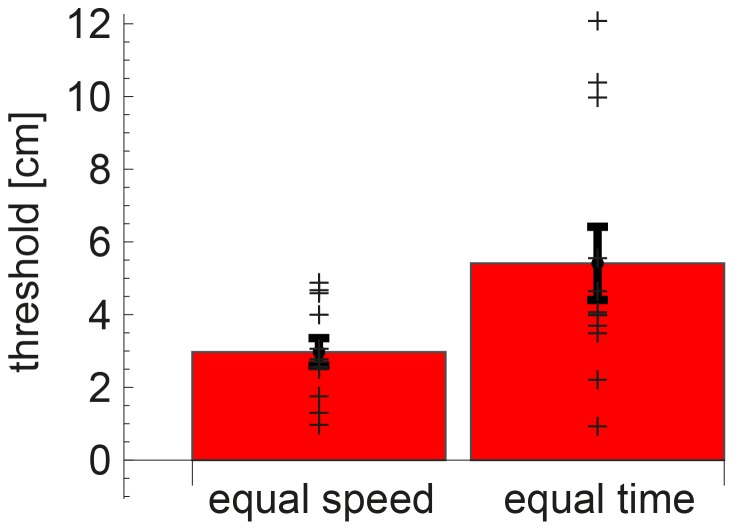
Thresholds for the passive condition, split into equal time and equal speed trials. The plusses mark the mean per participant, the bars show the mean over participants and the error bars show the standard error of the mean over participants. No significant difference between the 2 groups was found, but some participants did show extremely high thresholds for the equal time group.

For the grouping into equal speed and time trials for the passive condition, thresholds seem a bit lower when the speed of the first and second movement was the same, compared to trials in which the movement time was the same (see Fig. 6). However, they were not significantly different (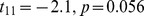).

**Figure 7:**
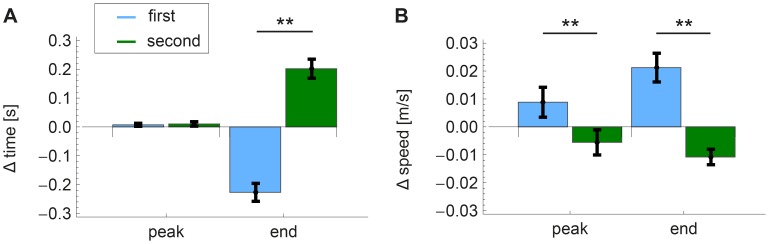
Position data, grouped according to answers of participants, with blue for ‘1’ and green for ‘2’. All parameters were calculated by subtracting the parameter of the first trial from that of the second trial. The bars show the mean over participants and the error bars show the standard error of the mean over participants. **A**. Time parameters, which are: time to peak speed and total movement time. **B**. Speed parameters, which are: peak speed and end speed. ** 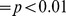

The velocity profiles that were constructed from the position data (for a typical example, see Fig. 3) showed quite some differences between the cases in which participants had answered ‘1’ and cases in which they had answered ‘2’ to be the longer distance. These differences are shown in Fig. 7. For the time parameters, there was a significant difference for end time (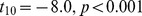), but the time of peak speed was not significantly different. For end time, the time difference was negative when participants answered ‘1’ and positive when they answered ‘2’, meaning that the time of the first trial was longer than that of the second when they answered ‘1’ and shorter when they answered ‘2’. For both speed parameters, there were significant differences between the two answers, which were 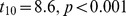 for peak speed and 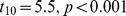 for end speed. Both speed differences were positive when participants answered ‘1’ and negative when they answered ‘2’, meaning that the speed was lower for the first stimulus of a trial than for the second stimulus when participants answered ‘1’, while it was higher when they answered ‘2’.

## Discussion

From the discrimination experiments, an influence of reference distance and perception mode on the precision of haptic distance perception was observed (see Fig. 4). Below, the results for each research question will be discussed in more detail. For each question, the possible implications of our findings for the design of haptic devices will also be discussed. However, these implications are only valid when optimizing for precision of distance perception.

### Reference distance

The discrimination threshold was influenced by reference distance. From Weber's law [45], a constant Weber fraction (and thus an increasing absolute threshold with increasing reference distance) could be expected. However, the Weber fraction was slightly higher for a reference distance of 15 cm than for the other two distances (Fig. 5). This is in line with results for the finger-span method, which also show an increasing Weber fraction for very small distances [10], [11], [19]. Probably, a whole arm movement is not the most efficient way to estimate a distance of 15 cm. This distance is only a bit longer than the span of one hand, so moving over this distance generates only a small difference in joint angles. For the two largest movement distances, a Weber fraction of about 11% was found. Thresholds for the finger-span method for intermediate distances were at least twice as small, so it is easier to precisely perceive distances by perceiving distance between thumb and forefinger, than by whole arm movements. To allow users of haptic devices to grasp objects between thumb and forefinger, the interfaces should be designed in such a way that operators can use their individual fingers and can receive feedback on them. When this is possible, operators can use the finger-span method to perceive object size for small distances. This would increase their precision, compared to using the movement method with a handle held in a power grip.

### Movement axis

We found no effect of movement axis on the precision of distance perception, while there is a well-known effect of movement axis on the accuracy of distance perception, called the radial-tangential illusion [26]. Generally, the distance of a radial movement is overestimated, compared to a tangential one. However, a difference in accuracy does not automatically imply a difference in precision. Imagine, for instance, that a participant perceives the same physical distance (e.g. 10 cm) as twice as large along the radial axis (20 cm), compared to the tangential axis (10 cm). This participant might perceptually also need a twice as large difference between 2 distances presented along the radial axis (e.g. 2 cm) to perceive them as being different, compared to 2 distances presented along the tangential axis (1 cm). However, the thresholds are measured in the physical world, so for this participant no difference in precision between the two directions will be found (both 1 cm). For more details on this topic, see the review by Ross [46]. Our measured discrimination thresholds did not differ between the cardinal axes, so we found no indication of an influence of the radial-tangential illusion on the precision of distance perception. As visual perception of depth (along the radial axis) is generally less precise than visual perception in the fronto-parallel plane (along the tangential and vertical axes [47]), haptic depth cues could potentially aid in precise perception of distances using devices that combine haptic and visual information.

### Movement mode

For the comparison between movement modes, we found a deterioration of precision for the passive condition, which is in line with the results on accuracy of distance perception [3]. This is an interesting observation, because it suggests that the perception of distance is not solely based on the position of the start and end points, but the way in which the movement in between the positions is made also seems to add information. The grouping of the data set into trials with the same speed and the same movement time (see Fig. 6) was made to assess whether participants are more likely to have been using speed differences or time differences in this task. Evidence from this grouping is not conclusive, as no significant differences were found, but it suggests that some participants were relying more on time cues than on speed cues, judged from extremely high thresholds for some participants in the equal time group. This finding suggests that taking away authority from the operator, for instance by providing strong guidance forces in haptic devices, could deteriorate precision of distance perception.

### Stimulus type

We found no effect of stimulus type. Although the thresholds are not significantly different, they do look a bit higher for the real conditions than for the rendered one. Usually, differences between stimulus types are in favor of real stimuli (e.g. [43]). Both the difference in hand posture (sliding a finger over a tube in the conditions using real stimuli and holding a handle in the conditions using rendered ones) and the difference between the stimulus properties (real and rendered) could be the cause of this. We suspect that the stimulus properties might be important, because the movement with the real stimulus was a bit less constrained than the movement with the rendered stimulus. Because the tubes were round, the carriage also had a little freedom to rotate around the tube, which could have resulted in a forward-backward movement. In the conditions using rendered stimuli, the maximum movement amplitude in this direction was 2 mm, which was the width of the haptic tunnel. For the conditions using real stimuli, position data were used to calculate the maximum movement amplitude in this direction per trial, which yielded a mean amplitude maximum of 5.8 mm over all trials. This could explain why the thresholds for the real condition look a bit higher. However, it is intriguing that the addition of cutaneous information, which was done in condition 8, did not seem to help the participants. From an optimal cue combination perspective [48], at least a little bit of improvement should be expected. It seems therefore that in our task, cutaneous information was so unreliable that it did not add much as a predictor in the cue combination model. Bergmann Tiest et al. [14] show that purely cutaneous distance discrimination is possible, but is a lot less precise than kinaesthetic perception. In their experiment, which was a passive perception experiment, there was also hardly any added value of combining cutaneous and kinaesthetic information. In our active case, the same principle seems to hold. This information seems to imply that for haptic devices it is not necessary to render surfaces that resemble real surfaces to ensure precise distance perception.

### Movement strategy

The grouping of the passive data into equal speed and equal time trials did not yield significant differences. However, the trend seems to suggest that some participants were more likely to use an estimate of the movement time than an estimate of movement speed as a strategy to find out which distance was the longer. For the active conditions, participants could use their own movement strategy to obtain the best estimate of movement distance. To get an insight into the strategy that the participants were using in this active case, the position data were analyzed (Fig. 7).

For both speed and time data significant differences between answering ‘1’ and ‘2’ were found. This can be understood by looking at the data qualitatively (for an example of the shape of the curves, see Fig. 3), as the velocity profiles of the movements within one trial look very much alike. It therefore seems that participants tried to reproduce the speed profile of the first trial during the second trial. This would be a smart strategy, as simply judging whether the end point is reached before or after the reproduced profile is finished, would give the participants all the information they need to answer the question. If we assume that this was indeed the strategy that participants were using, we can speculate on its effect on the parameters. For the time data (shown in Fig. 7A), the parameters are congruent with our explanation, as the peak times did not differ, while the end times did. When participants answered ‘1’, the sign of the end time difference was negative, which means that the first trial took longer when they judged it to be the longer distance. For the speed data (shown in Fig. 7B), both parameters differed significantly between the answers. For end speed, the sign makes sense: when participants answered ‘1’, the sign of the end speed difference was positive, meaning that the speed in the second trial was still higher at the end. This indicates that participants could not complete their profile in the second trial and thus judged this distance to be the shorter one. For peak speed, however, a significant difference was also found, so the reproduction of the speed profiles was not perfect. When, for instance, the peak speed was higher during the second trial, this would result in a smaller end time and a higher end speed in the second trial, which would induce participants to judge the first distance to be the longer. This is indeed what the parameters reflect: when the peak speed in the second trial was higher, the participants answered ‘1’ more often. So, this imperfect reproduction of the peak speed seems to have influenced the perception of the participants.

Concluding, the parameters can be explained by assuming that the participants tried to reproduce the speed profile of the first trial during the second trial and based their decision on whether they could complete their profile or not. Apparently, they succeeded in reaching the peak speed at the same moment, but they did not succeed in reaching exactly the same peak speed magnitude. Of course, this reasoning is based on speculation, but it does explain all the measured parameters.

## Conclusion

Overall, we found that for movements along distances of 25 and 35 cm, a Weber fraction of about 11% was reached for the precision of active haptic distance perception along all cardinal axes. Passive movements worsen the precision, while adding cutaneous information does not improve it.
